# Supplementary material for: Accessible chemical space for metal nitride perovskites
Source: Chem Sci. 2023 Aug 15;14(34):9175–85. doi: 10.1039/d3sc02171h (PMC10466337; doi:10.1039/d3sc02171h)
Supplement: SC-014-D3SC02171H-s001 [file SC-014-D3SC02171H-s001.pdf]

Electronic Supplementary Material (ESI) for Chemical Science.
This journal is © The Royal Society of Chemistry 2023

Supplemental Information: Accessible Chemical Space for Metal Nitride Perovskites

Bastien F. Grosso,† Daniel W. Davies,† Bonan Zhu,† Aron Walsh,*,‡ and David O. Scanlon*,†

†*Department of Chemistry, University College London, London, United Kingdom*

‡*Department of Materials, Imperial College London, London, United Kingdom*

E-mail: a.walsh@imperial.ac.uk; d.scanlon@ucl.ac.uk

We present in Fig. S1 to Fig. S8, the effect of the 15 octahedral tilts on the energy of each of the 279 candidates, after one loop of electronic relaxation (dashed lines) and after full relaxation of the ions and lattice (continuous lines).

In Fig. S9 to Fig. S13, we present, for each of the 25 closed-shell candidates, the electronic band structure and density of states for the (A) and (G) structures as well as their respective crystal structures.

Table 1 and 2, contain the convergence parameters for the 25 candidates presented in the main text and for the 86 competing phases used to compute the energy hull, respectively. The corresponding structure can be found in the data repository provided (`https://github.com/SMTG-UCL/High_throughput_search_ABN3`). Finally, the list of the pseudopotentials used is presented in Table 3.

Table 1: Convergence parameters of the 25 candidates presented in the main text.

	**Glazer**		**AIRSS**	
	**Encut**	**k-mesh**	**Encut**	**k-mesh**
$GaMoN_3$	450	3x5x4	450	5x8x3
$GaWN_3$	450	6x6x5	450	11x6x4
$HfNbN_3$	450	4x3x2	450	9x4x2
$HfSbN_3$	450	5x5x3	450	9x5x3
$HfTaN_3$	500	4x3x2	500	5x4x3
$HfVN_3$	450	4x5x4	450	9x4x2
$InMoN_3$	550	4x5x6	550	5x2x3
$InWN_3$	450	4x5x4	450	7x7x7
$LaMoN_3$	450	5x7x6	450	7x7x6
$LaWN_3$	450	7x7x7	450	7x7x2
$ScMoN_3$	450	6x6x4	450	9x4x3
$ScWN_3$	450	4x5x6	450	9x4x3
$SnNbN_3$	450	5x6x4	450	11x5x3
$SnSbN_3$	450	5x5x4	450	7x7x7
$SnTaN_3$	450	5x5x4	450	5x4x4
$SnVN_3$	450	5x5x4	450	4x4x3
$TiVN_3$	450	2x5x5	450	9x5x4
$TlMoN_3$	550	4x5x6	550	3x5x5
$TlWN_3$	450	6x6x6	450	9x6x3
$YMoN_3$	450	5x6x6	450	5x5x4
YWN_3	450	5x5x6	450	4x4x4
$ZrNbN_3$	450	4x4x4	450	9x5x4
$ZrSbN_3$	450	5x5x4	450	5x3x4
$ZrTaN_3$	450	4x4x3	450	5x4x5
$ZrVN_3$	450	2x6x6	450	9x4x3

Table 2: List of the competing phases and convergence parameters. The energy cutoff (Encut) and k-mesh grid (k-mesh) are indicated next to each materials.

**Material**	**Encut**	**k-mesh**	**Material**	**Encut**	**k-mesh**	**Material**	**Encut**	**k-mesh**
Ga_1N_1	500	7x7x7	N_4	750	8x8x6	Tl_3	400	11x11x11
Ga_2Mo_6	400	8x8x8	N_8	750	9x9x9	Tl_{29}	400	6x6x6
Ga_2N_2	450	8x8x5	N_8	750	9x9x9	V_1	500	17x17x17
Ga_4	400	6x6x6	Nb_1	400	17x17x17	V_1N_1	500	9x9x9
Ga_4	350	12x12x11	Nb_1N_1	500	10x10x8	V_4Sn_8	400	6x7x6
$Ga_{41}Mo_8$	400	5x5x5	Nb_4Sn_8	350	8x8x8	V_6N_3	450	5x5x5
$Ga_{62}Mo_{12}$	400	4x4x2	Nb_6N_3	450	6x6x5	V_6Sn_2	450	9x9x9
Hf_1N_1	500	11x11x11	Nb_6Sn_2	400	10x10x10	V_16N_2	450	4x4x10
Hf_2	450	11x11x6	$Nb_{10}N_{12}$	500	7x7x3	W_1	450	16x16x16
Hf_3N_2	450	8x8x8	$Nb_{12}Sn_{10}$	400	3x3x5	W_4N_6	750	6x6x5
Hf_4N_2	500	5x5x11	Sb_2	300	11x11x11	Y_1N_1	550	8x8x8
Hf_4Sb_4	300	11x11x11	Sc_1N_1	450	9x9x9	Y_2	400	18x18x10
Hf_6Sb_6	350	7x7x2	Sc_2	450	9x9x5	Y_3	400	13x13x13
Hf_8Sb_{16}	300	12x4x2	Sn_1Sb_1	450	15x15x15	Y_4	400	9x9x3
$Hf_{10}Sb_{18}$	350	4x4x4	Sn_2	400	11x11x11	$Y_4W_4N_{12}$	450	4x4x4
$Hf_{12}N_{16}$	500	5x5x2	Ta_1	450	11x11x11	Zr_1N_1	550	16x16x16
$Hf_{12}Sb_4$	350	5x5x4	Ta_3N_3	700	7x7x11	Zr_2	450	16x16x9
$Hf_{20}Sb_{12}$	350	3x3x3	Ta_4Sn_8	450	10x10x10	Zr_4N_2	450	5x5x11
In_1N_1	500	8x8x8	Ta_6N_3	450	9x9x8	Zr_4Sb_2	450	7x7x9
In_2N_2	500	8x8x4	Ta_6N_{10}	700	7x7x3	Zr_4Sb_4	400	10x10x10
In_3	450	14x14x14	Ta_6Sn_2	450	10x10x10	Zr_6Sb_6	400	9x9x3
La_1	350	16x16x16	Ta_{30}	400	4x4x6	Zr_8Sb_{16}	400	7x3x2
La_2N_2	550	7x7x4	Ta_{30}	400	4x4x6	$Zr_{10}Sb_6$	400	4x4x5
$La_2W_2N_6$	450	5x5x5	Ti_1N_1	450	16x16x16	$Zr_{12}N_{16}$	550	7x3x3
La_4	350	13x13x4	Ti_2	400	9x9x5	$Zr_{12}Sb_4$	450	7x7x4
$La_4Mo_4N_{12}$	750	2x2x3	Ti_3	400	7x7x11	$Zr_{20}Sb_{12}$	400	4x3x3
Mo_1	400	17x17x17	Ti_4N_2	450	7x7x11	$Zr_{22}Sb_{36}$	300	4x4x1
Mo_4N_2	450	6x6x8	Tl_1	400	24x24x24	$Zr_{28}Sb_{16}$	300	2x3x3
Mo_8N_8	550	6x6x6	Tl_2	400	16x16x8			

Table 3: List of pseudopotentials used for each atom.

PAW_PBE Ga_d 06Jul2010
PAW_PBE Mo_pv 04Feb2005
PAW_PBE N 08Apr2002
PAW_PBE W_sv 04Sep2015
PAW_PBE Hf_pv 06Sep2000
PAW_PBE Nb_pv 08Apr2002
PAW_PBE Sb 06Sep2000
PAW_PBE Ta_pv 07Sep2000
PAW_PBE V_pv 07Sep2000
PAW_PBE In_d 06Sep2000
PAW_PBE La 06Sep2000
PAW_PBE Sc_sv 07Sep2000
PAW_PBE Sn_d 06Sep2000
PAW_PBE Ti_pv 07Sep2000
PAW_PBE Tl_d 06Sep2000
PAW_PBE Y_sv 25May2007
PAW_PBE Zr_sv 04Jan2005

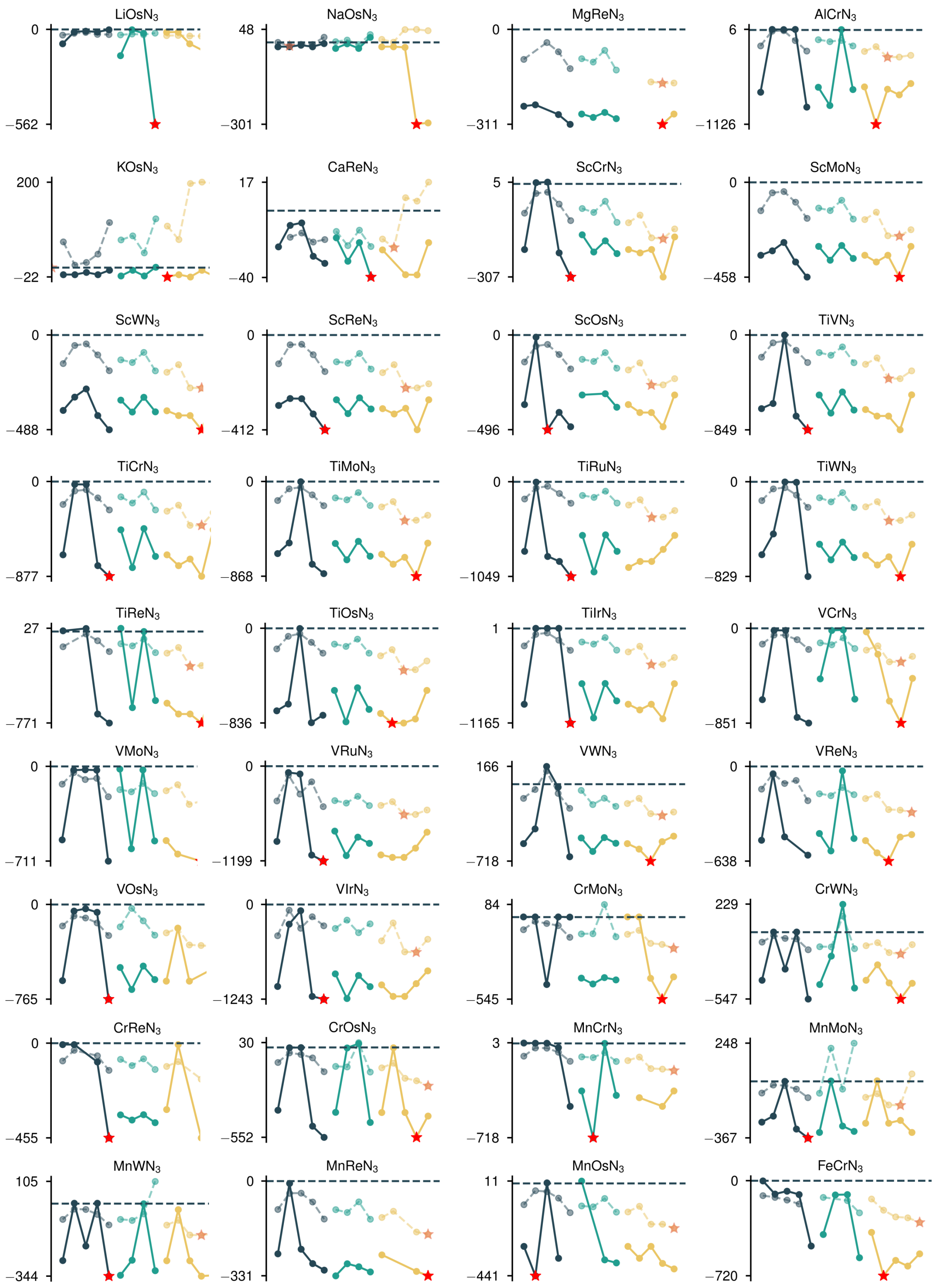

Figure S1: Energy of tilts for fixed ions (dashed lines) and full relaxation (continuous lines)

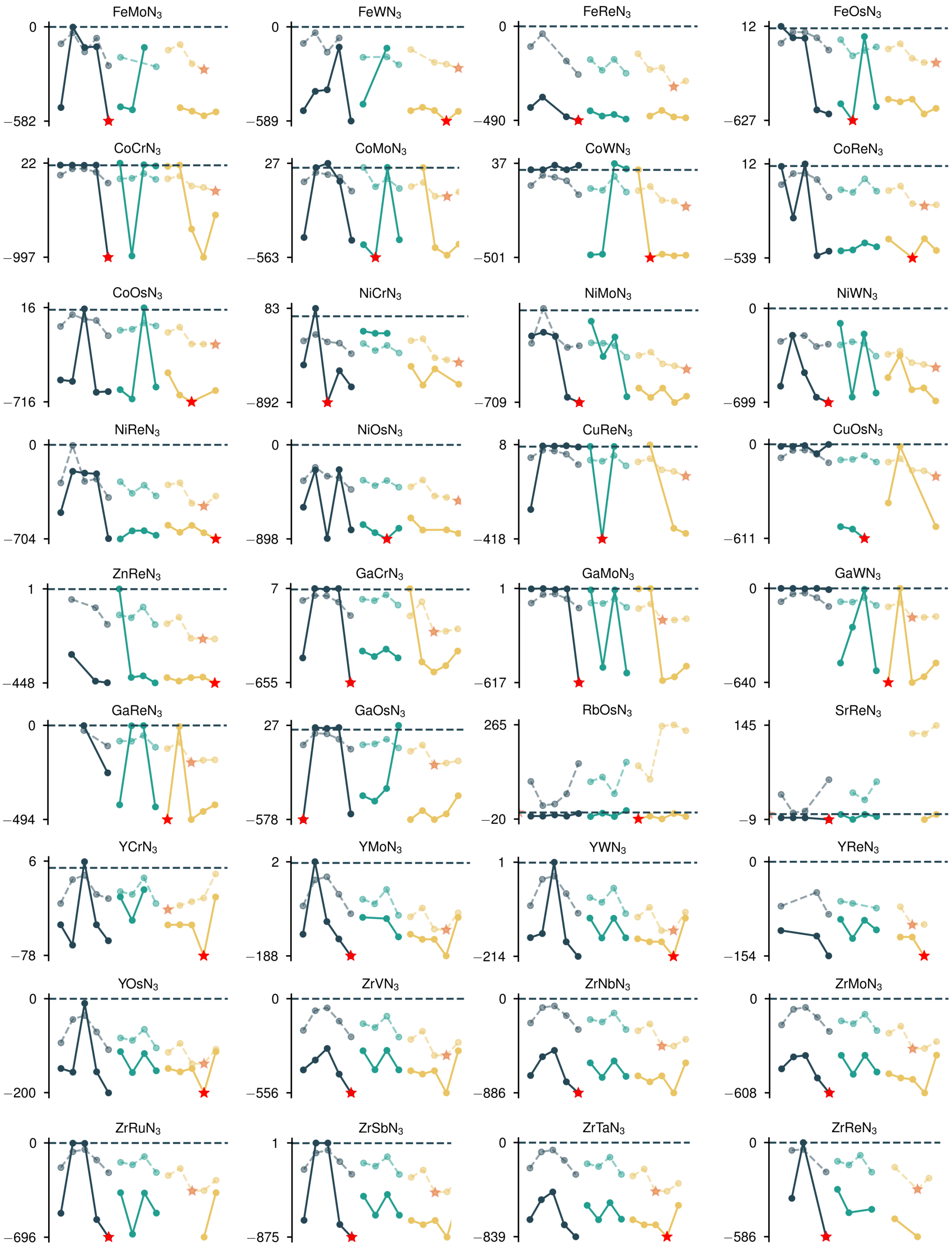

Figure S2: Energy of tilts for fixed ions (dashed lines) and full relaxation (continuous lines)

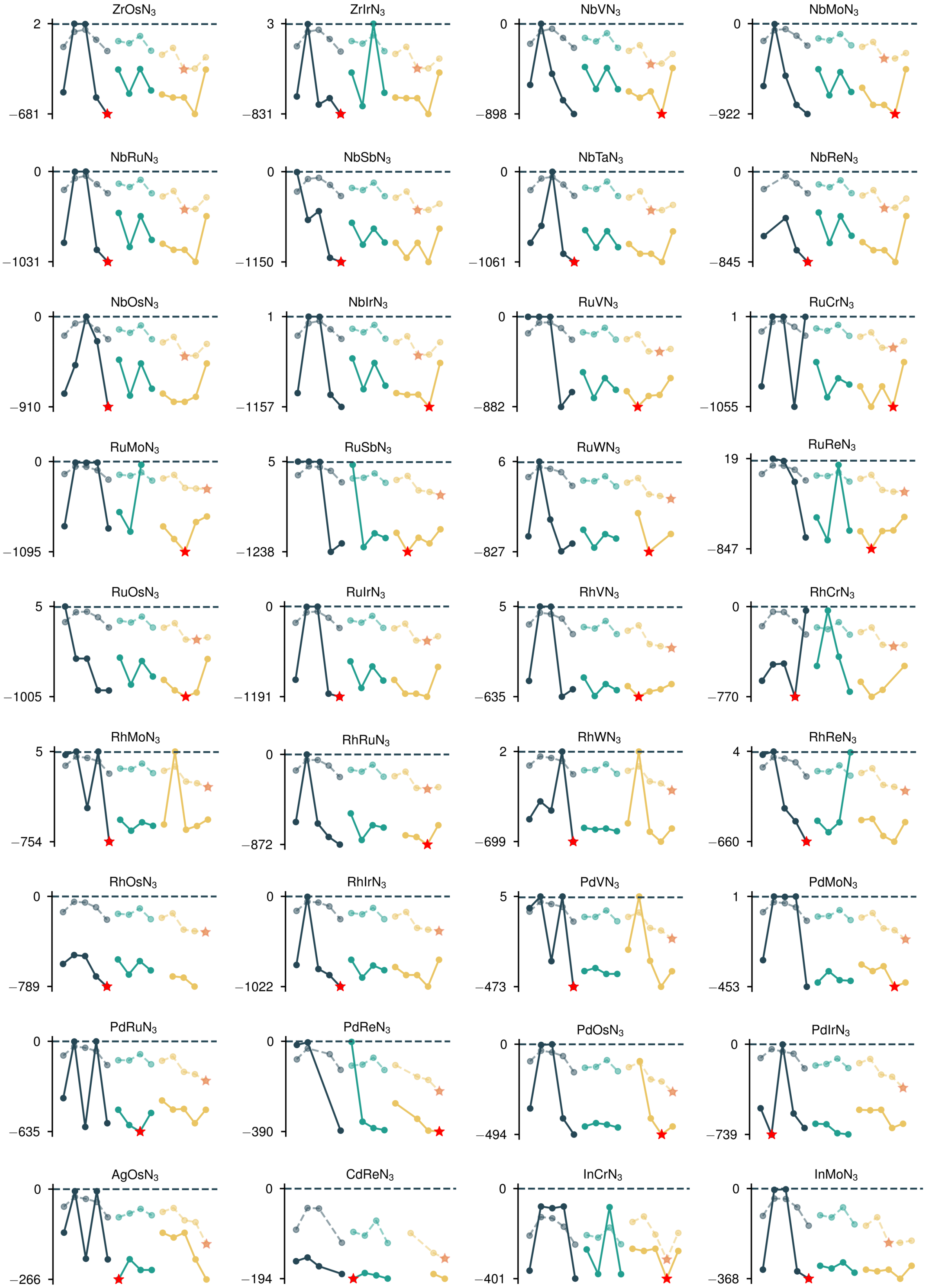

Figure S3: Energy of tilts for fixed ions (dashed lines) and full relaxation (continuous lines)

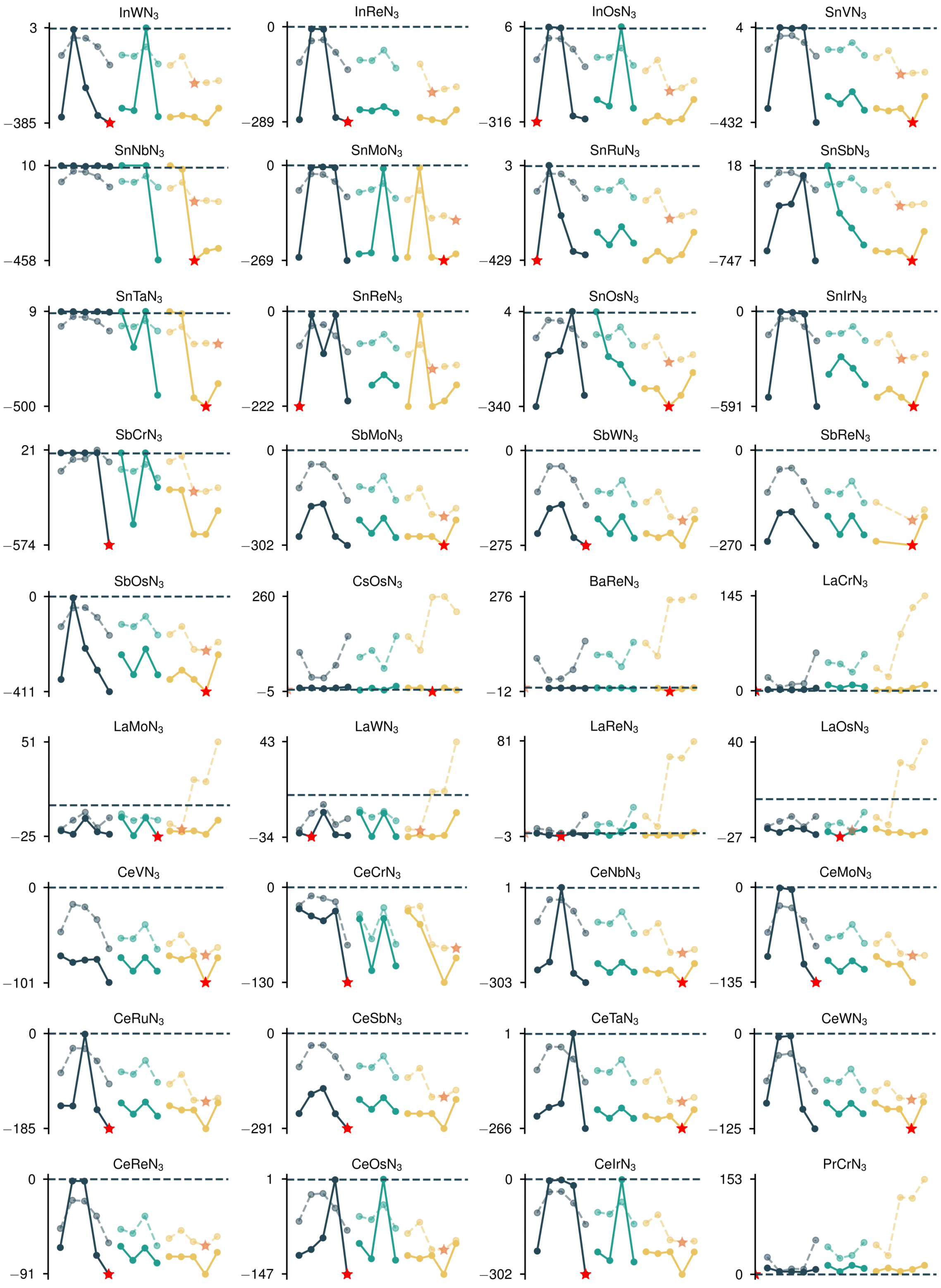

Figure S4: Energy of tilts for fixed ions (dashed lines) and full relaxation (continuous lines)

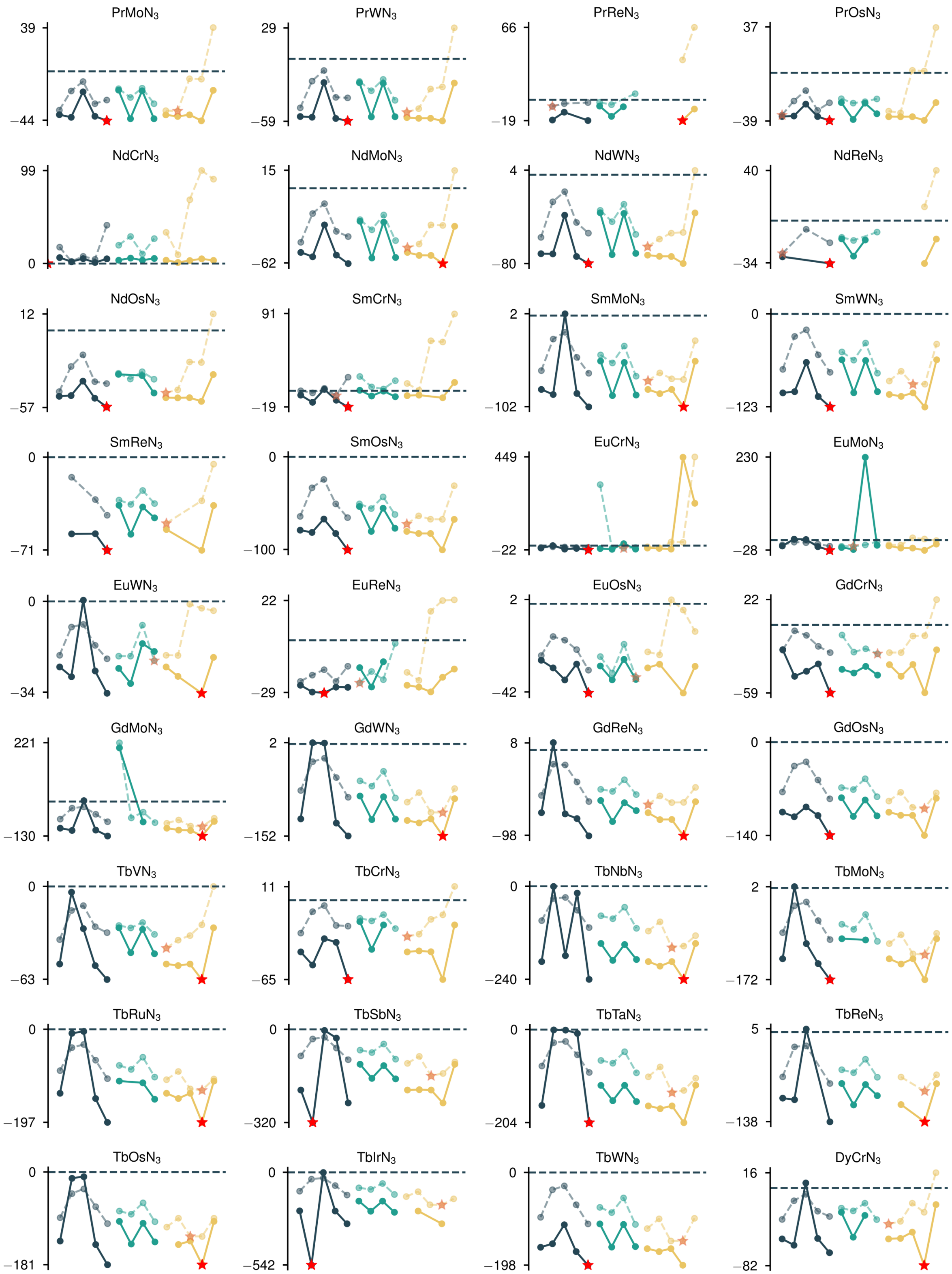

Figure S5: Energy of tilts for fixed ions (dashed lines) and full relaxation (continuous lines)

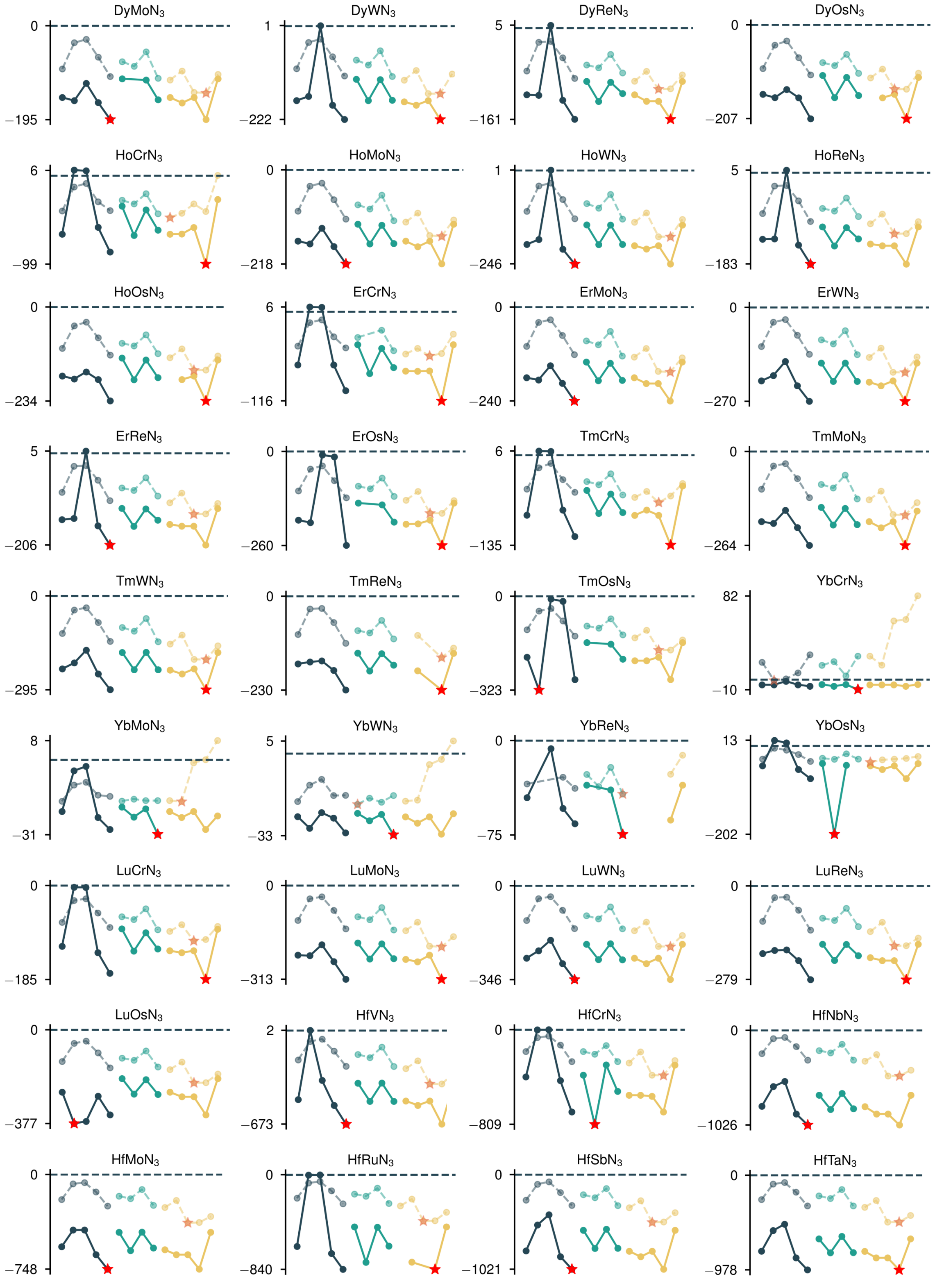

Figure S6: Energy of tilts for fixed ions (dashed lines) and full relaxation (continuous lines)

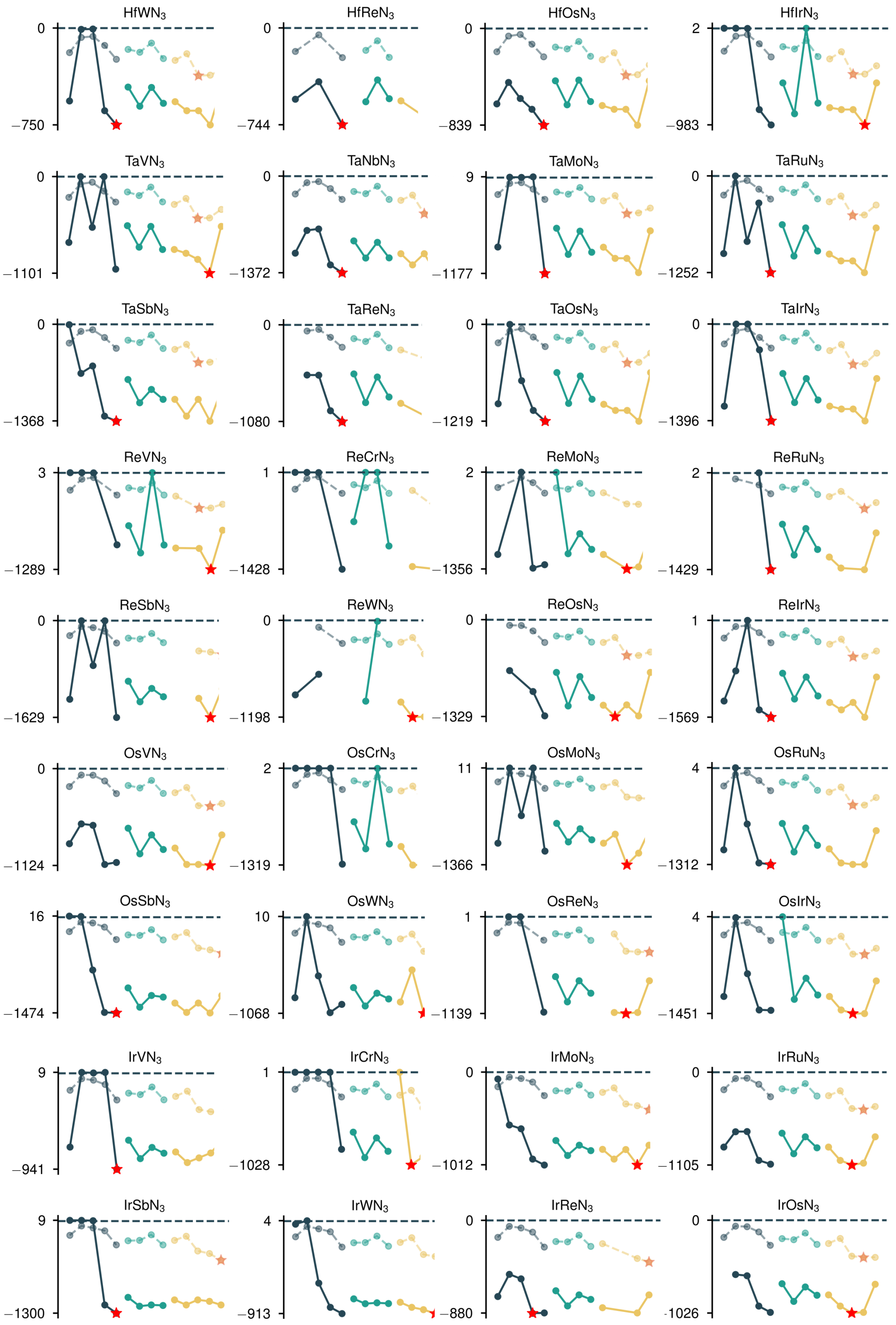

Figure S7: Energy of tilts for fixed ions (dashed lines) and full relaxation (continuous lines)

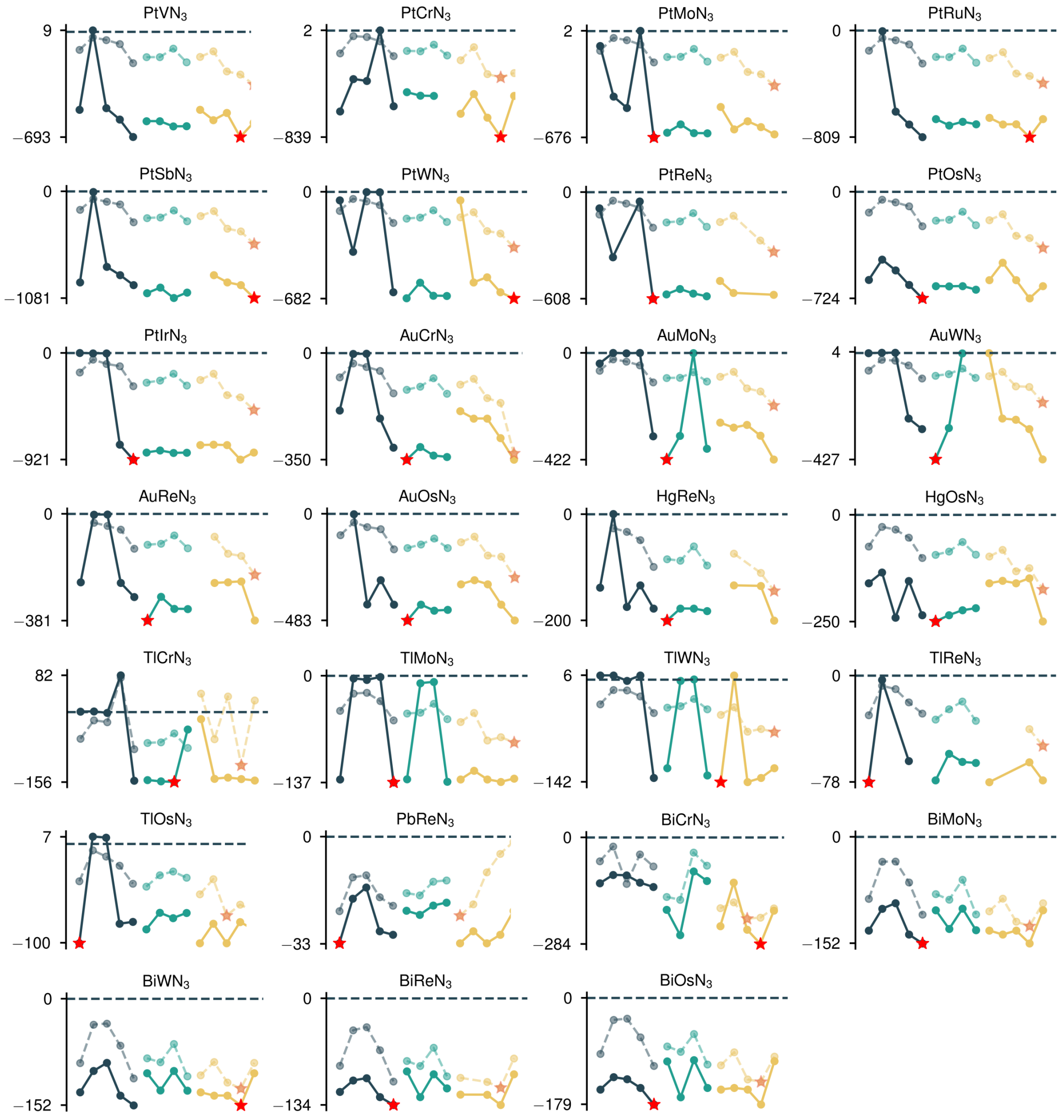

Figure S8: Energy of tilts for fixed ions (dashed lines) and full relaxation (continuous lines)

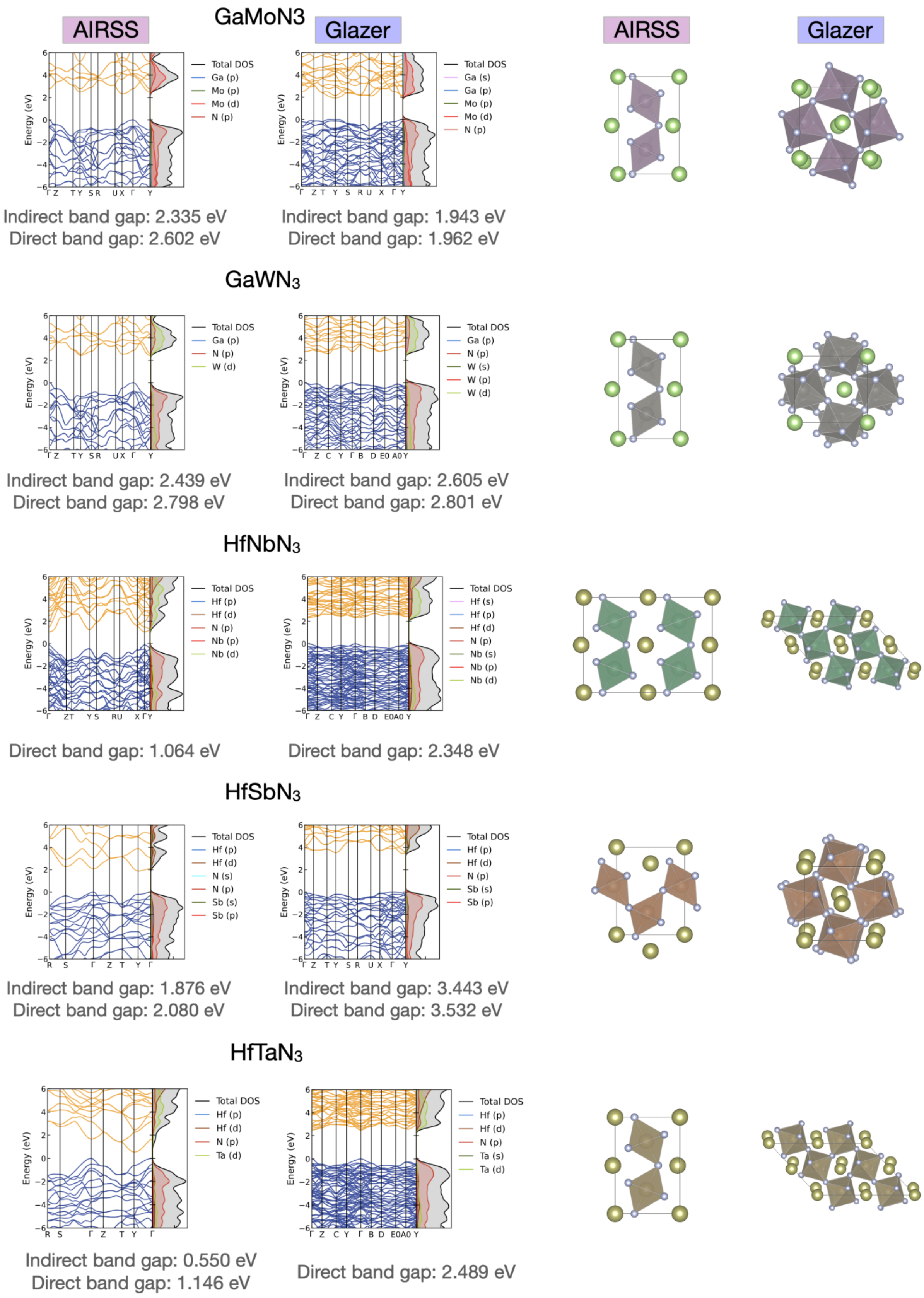

Figure S9: Electronic structure, DOS and relative crystal structures.

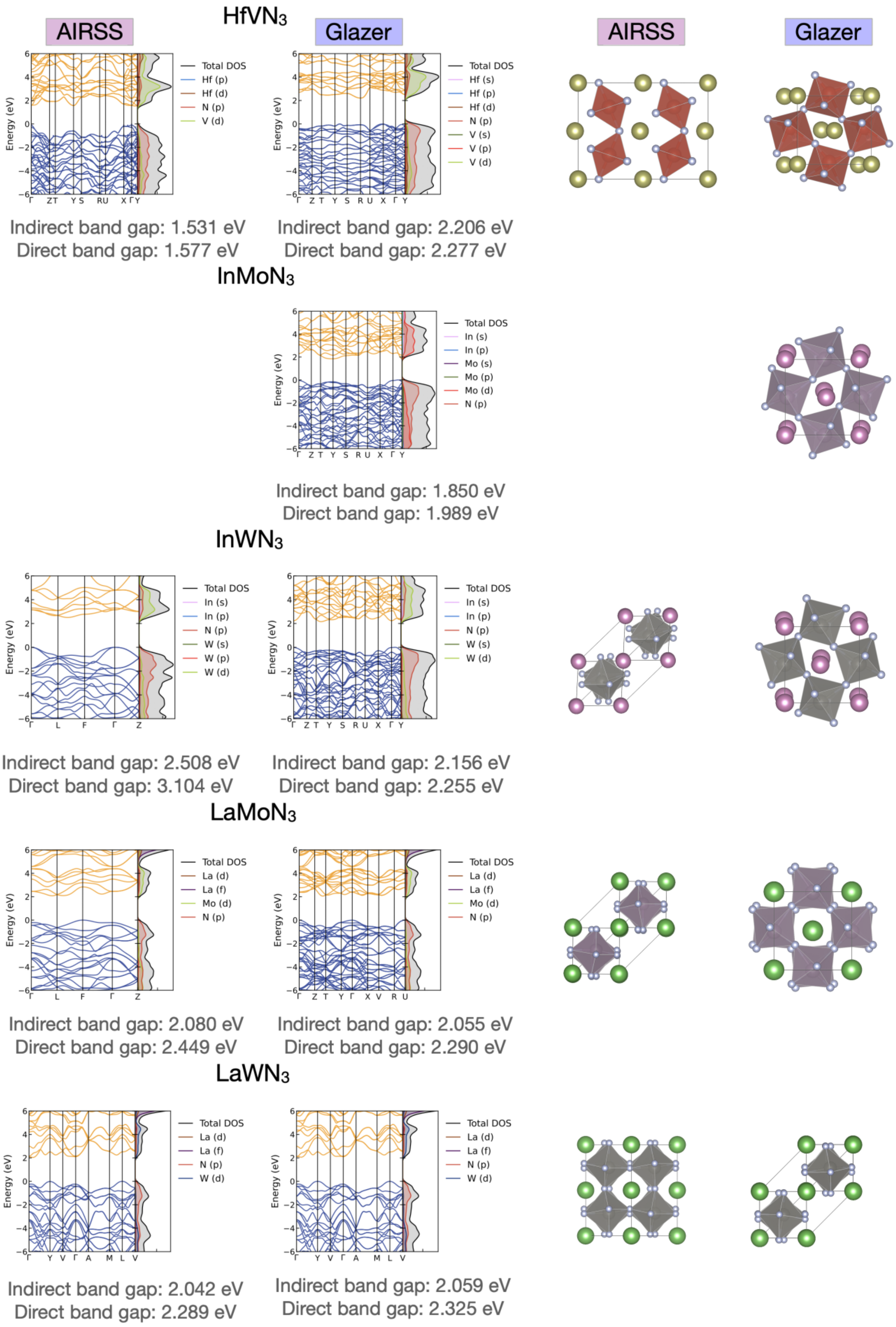

Figure S10: Electronic structure, DOS and relative crystal structures.

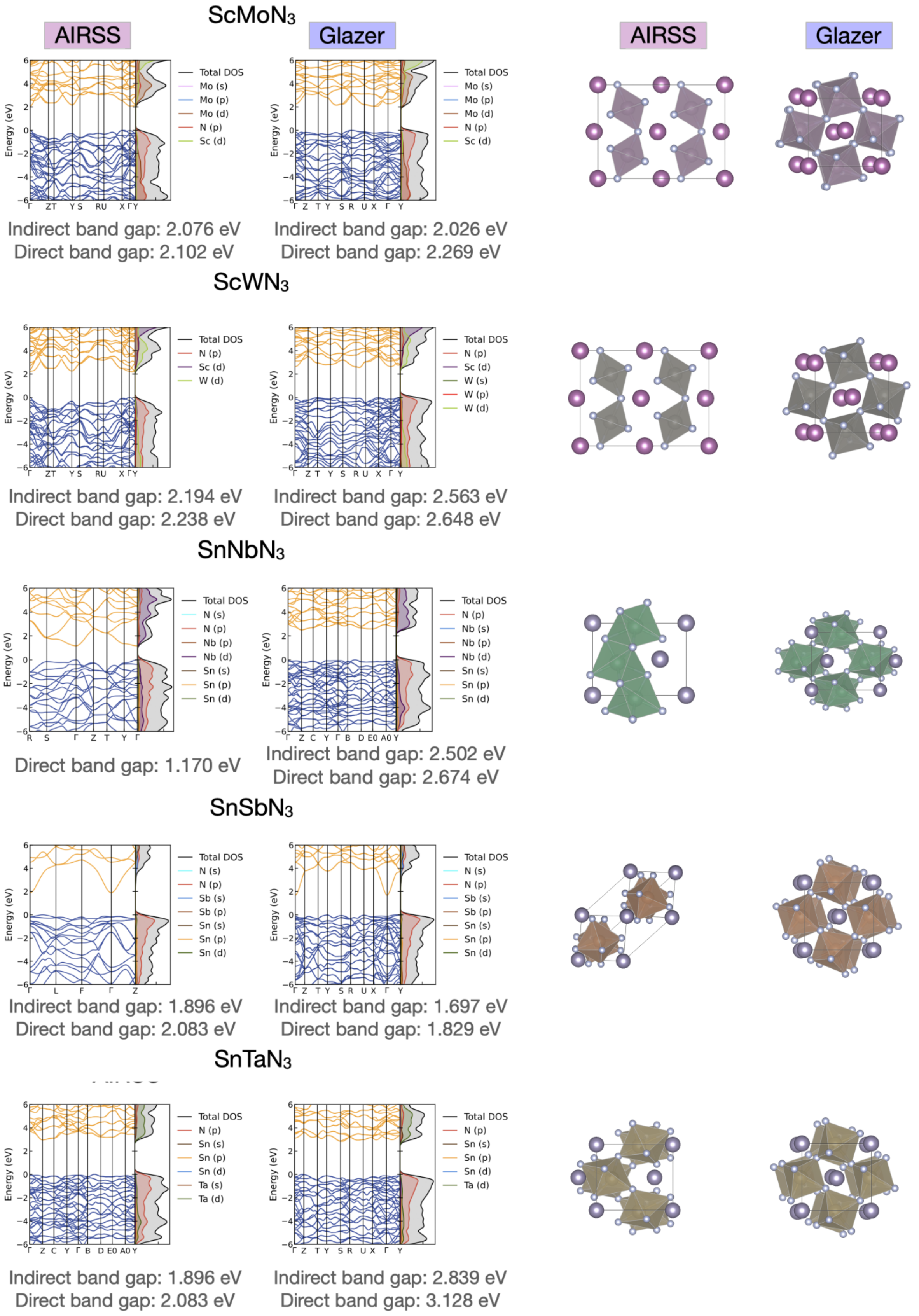

Figure S11: Electronic structure, DOS and relative crystal structures.

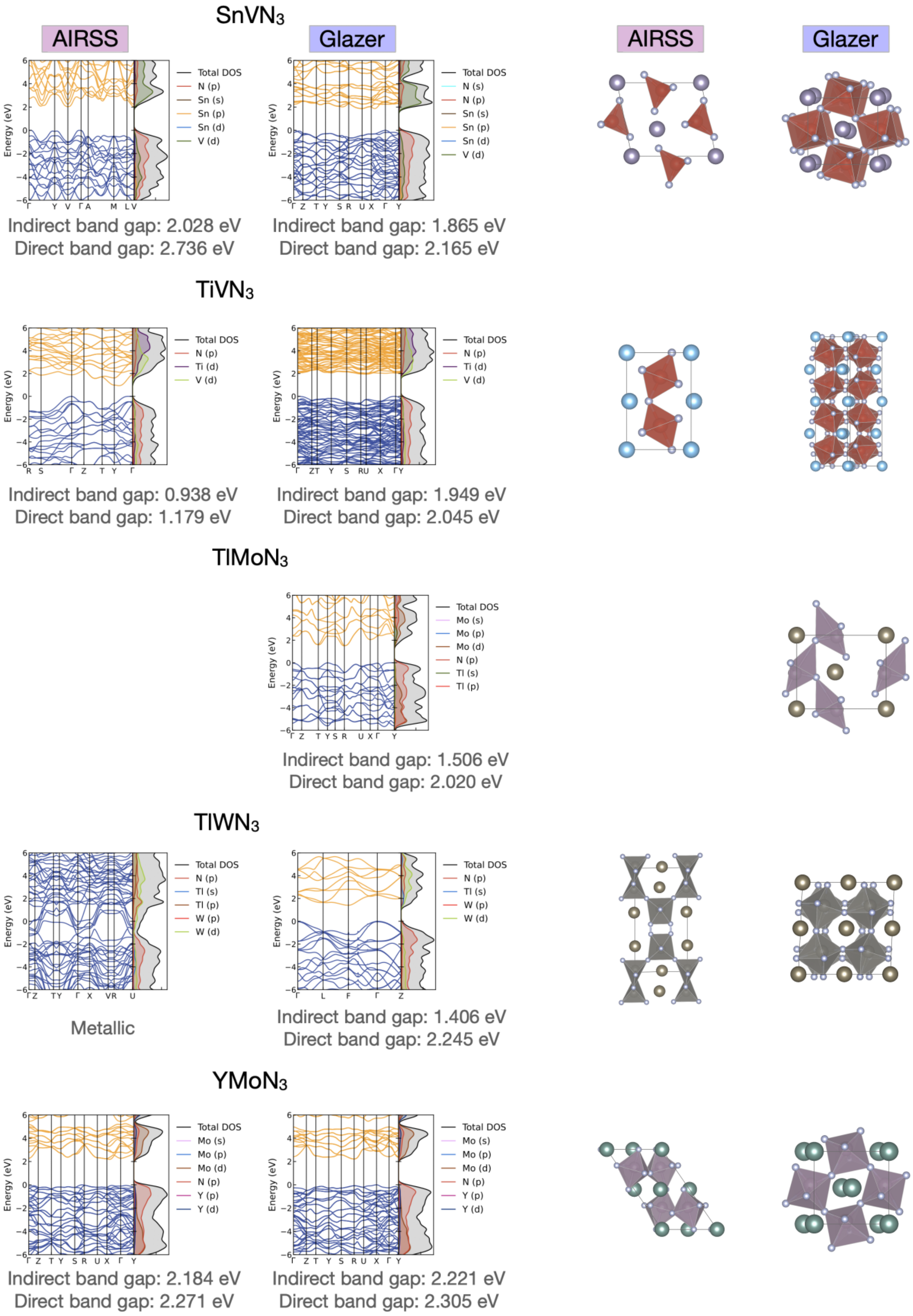

Figure S12: Electronic structure, DOS and relative crystal structures.

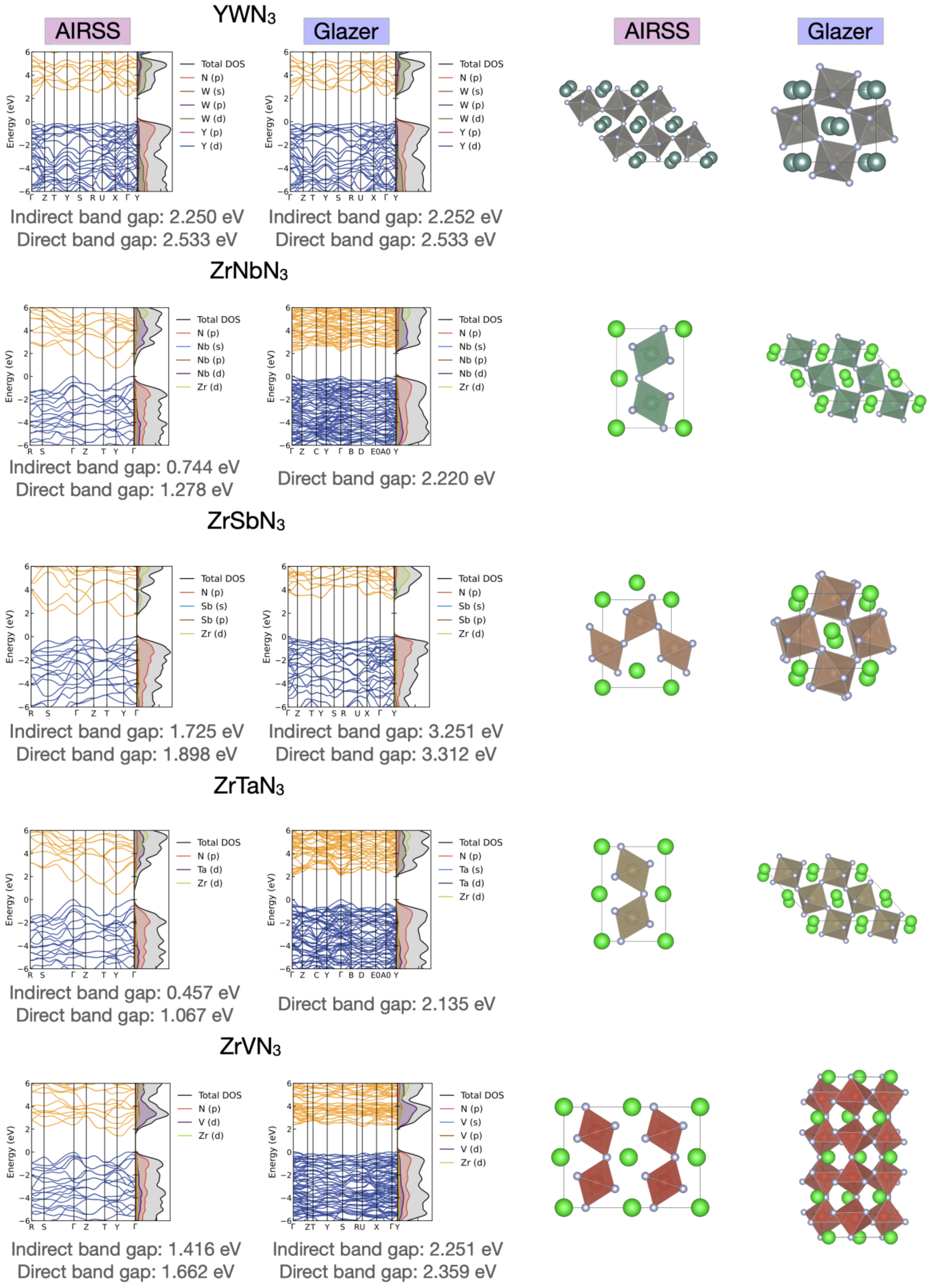

Figure S13: Electronic structure, DOS and relative crystal structures.
